# The Reward-Based Eating Drive Scale: A Self-Report Index of Reward-Based Eating

**DOI:** 10.1371/journal.pone.0101350

**Published:** 2014-06-30

**Authors:** Elissa S. Epel, A. Janet Tomiyama, Ashley E. Mason, Barbara A. Laraia, William Hartman, Karen Ready, Michael Acree, Tanja C. Adam, Sachiko St. Jeor, David Kessler

**Affiliations:** 1 Department of Psychiatry, University of California San Francisco, San Francisco, California, United States of America; 2 UCSF Center for Obesity Assessment, Study, & Treatment, San Francisco, California, United States of America; 3 Department of Psychology, University of California Los Angeles, Los Angeles, California, United States of America; 4 UCSF Osher Center for Integrative Medicine, San Francisco, California, United States of America; 5 School of Public Health, University of California, Berkeley, California, United States of America; 6 MD Weight Management Program, San Francisco, California, United States of America; 7 Department of Human Biology, Maastricht University, Maastricht, Netherlands; 8 Division of Endocrinology Nutrition and Metabolism, University of Nevada School of Medicine, Reno, Nevada, United States of America; 9 Department of Pediatrics, UCSF School of Medicine, San Francisco, California, United States of America; University of Southern California, United States of America

## Abstract

Why are some individuals more vulnerable to persistent weight gain and obesity than are others? Some obese individuals report factors that drive overeating, including lack of control, lack of satiation, and preoccupation with food, which may stem from reward-related neural circuitry. These are normative and common symptoms and not the sole focus of any existing measures. Many eating scales capture these common behaviors, but are confounded with aspects of dysregulated eating such as binge eating or emotional overeating. Across five studies, we developed items that capture this reward-based eating drive (RED). Study 1 developed the items in lean to obese individuals (n = 327) and examined changes in weight over eight years. In Study 2, the scale was further developed and expert raters evaluated the set of items. Study 3 tested psychometric properties of the final 9 items in 400 participants. Study 4 examined psychometric properties and race invariance (n = 80 women). Study 5 examined psychometric properties and age/gender invariance (n = 381). Results showed that RED scores correlated with BMI and predicted earlier onset of obesity, greater weight fluctuations, and greater overall weight gain over eight years. Expert ratings of RED scale items indicated that the items reflected characteristics of reward-based eating. The RED scale evidenced high internal consistency and invariance across demographic factors. The RED scale, designed to tap vulnerability to reward-based eating behavior, appears to be a useful brief tool for identifying those at higher risk of weight gain over time. Given the heterogeneity of obesity, unique brief profiling of the reward-based aspect of obesity using a self-report instrument such as the RED scale may be critical for customizing effective treatments in the general population.

## Introduction

The fundamental causes of obesity are complex and linked to family history, genetic predispositions, and their interaction with the toxic food environment [1]. There is a striking differential in vulnerability to obesity that has largely eluded researchers. Once obese, many individuals attempt some form of weight loss [2], but most weight loss programs, whether pharmacological or behavioral, fail to promote long-term maintenance of weight loss [3]. While a small subset of people succeed in long-term weight loss, most obese individuals lose less weight than desired, gain much of it back within a year, and often undergo repeated cycles of weight loss and regain [4], [5]. We can therefore categorize some individuals according to both persistence of, and heightened vulnerability to, obesity.

There are clues to this most resistant problem, if one listens carefully to the daily-life experiences of individuals who have persistent struggles with weight gain. Many report an excessive drive to eat characterized by a trio of complaints – feeling a lack of control over eating, eating rapidly without experiencing satisfaction or satiation, and preoccupation with thoughts about food. These strong drives tend to be in response to the natural reward of highly palatable foods (foods high in fat, sugar, and salt). This trio, which emerged from clinical phenomenology of the common habitual and compulsive drive to overeat, was developed amidst extensive discussions with obese individuals, dieters, and clinicians, and has been expounded in the popular press under the label “conditioned hypereating” [6]. This construct of conditioned hypereating has been linked to palatable combinations of salt, sugar, and fat [6], [7]. Given the heterogeneity of the causes of obesity, it may be that this cluster of behavioral symptoms – excessive drive to eat that results from feelings of lack of control, diminished satiety, and preoccupation with food – that should be identified and treated, rather than targeting BMI alone. Here we focus on developing a brief measure to tap this normative but compulsive syndrome of strong eating drive.

These common aspects appear to reflect what might be called “reward-based eating”. Researchers have increasingly recognized the plausibility of reward-based eating as evidenced by both behavioral and neurobiological studies [8]–[15]. Reward-based eating can be problematic: it can override satiety signals, leading people to overeat beyond their caloric need. Calorie surpluses that result from this overeating, reinforced over time through shifts in the dopaminergic pathways that regulate neuronal systems associated with reward sensitivity [16], can foster excessive weight gain when the energy intake associated with the behavior is not compensated for by equal amounts of energy expenditure or previous/subsequent dietary restraint behaviors. Palatable food consumption and uncontrollable eating are now known to engage systems involved in reward processes such as the opioid and dopamine systems [17], and individuals at higher levels of Body Mass Index (BMI) are more sensitive to reward-related processes [18], [19].

As yet there are few self-report instruments that target these aspects of eating. The Power of Food Scale [20], [21] taps appetite for palatable food at three levels of food proximity (food available, food present, and food tasted); however, it is more narrowly focused on assessing the impact of food environments characterized by easily-accessible, highly-palatable foods. Despite recent advances in self-report assessment of normative eating behavior, we are unaware of a self-report instrument that targets the cluster of thoughts and behaviors characterizing reward-based eating that also correlates with weight or weight gain. Existing measures commonly used to measure eating behaviors such as binge eating, emotional eating, external eating, and dietary restraint, include the Three Factor Eating Questionnaire [22], the Dutch Eating Behavior Questionnaire [23], and the Binge Eating Scale [24]. While select items in some subscales from these measures appear to tap characteristics of reward-based eating, these larger scales lack subscales that specifically quantify it, thus offering merely an indirect index of reward-based eating behavior. In the current set of studies, we therefore attempt to capture basic, non-pathological eating drive characterized by the co-existence of the trio of common reward-related eating behavior complaints–lack of control, lack of satiation, and preoccupation with food.

At the very extreme, reward-based eating could lead to dependence on highly palatable food, food addiction and obesity. Prior research has predominantly focused on this type of clinically pathological eating, and most studies in this area have been designed to draw direct parallels between this type of eating and actual food dependence, or even food addiction. In rodent models, animals that are fed high-sugar, high-fat, or otherwise highly palatable food develop symptoms mirroring drug dependence criteria in the DSM-IV [10]. In humans, efforts to mimic tests investigating the abuse potential of drugs have succeeded using carbohydrates [25], and food- and drug-dependent individuals demonstrate similar brain response abnormalities such as changes in reward sensitivity through blunted response to consuming palatable food [26], [27], and decreased frontal cortical gray matter volume [28]. Gray matter volume generally increases in response to intensive training in various domains requiring focused attention, such as cognitive, sensory, and motor-based skills [28]–[31] and decreases at an accelerated rate among individuals with alcoholism, cocaine dependence, schizophrenia, and other psychological disorders [17], [32]–[34].

Because empirical work in this area has focused on characterizing extreme, pathological levels of eating related to reward processes, measurement approaches have focused on applying DSM-IV criteria to food [9], [12], [35]. Gearhardt and colleagues developed the Yale Food Addiction Scale (YFAS) [12] to measure addiction-like diagnostic criteria. Higher YFAS scores correlate with clinical co-morbidities, psychological risk factors, and motivation for food that mirror that of substance dependence [9]. Higher scores on the YFAS also correlate with greater activation in the same neural reward circuitry and pattern of activation as in substance dependence [26]. Individuals endorsing more YFAS symptoms show greater activation in the amygdala, anterior cingulate cortex, caudate, dorsolateral prefrontal cortex, and medial OFC when exposed to palatable food cues, such as pictures of milkshakes, relative to neural cues, such as glasses of water [36].

Although drawing direct connections between food addiction and drug addiction is valuable, so too is understanding, studying, and measuring basic reward-related eating behavior. Rather than focusing narrowly on food addiction, stepping back to examine the common phenomena of basic reward-based eating nets a number of advantages. First, reward-based eating behavior is likely highly prevalent, and exists on a continuum. The co-existence of the common reward-related eating behavior complaints – lack of control, lack of satiation, and preoccupation with food – likely occur long before any eating pathology develops, making it an intermediate phenotype that may be a more effective early target for intervention. Furthermore, most individuals who endorse reward-based eating will not necessarily progress to food addiction. A growing body of data have suggested prevalence rates for food dependence (as defined by the YFAS) to be approximately 11% in non-obese individuals [37] and approximately 15% in a sample of obese individuals enrolled in behavioral weight loss programs [13]. Recent data have also suggested that women with BMIs between 23.0 and 24.9 (i.e., normal weight) are approximately twice as likely as women with BMIs between 18.5 and 22.9 to report food addiction [38].

We therefore developed and tested a short self-report scale to use as a tool to measure reward-based eating drive (RED). We hypothesized that RED scores would 1) positively associate with BMI in adulthood; 2) positively associate with greater likelihood of onset of obesity in childhood (by self report); and 3) predict increases in BMI over time. Rather than a diagnostic-style measure of food dependence that captures the pathological end of the eating spectrum to parallel the criteria for drug addiction (i.e., YFAS), we conceptualize the RED scale as capturing variation in the non-pathological population. We drew on select items from pre-existing questionnaires and when necessary constructed new items intended to capture reward-based processes to create a brief, targeted, and clinically meaningful scale that would capitalize on a combination of novel, face-valid items and established, clinically meaningful items. We had experts review and rate each item to refine the final scale and we conducted psychometric testing across 3 samples.

## Methods

Study 1: All procedures were approved by the University of Nevada Institutional Review Board and all participants provided written informed consent. Study 2: All procedures were approved by the University of California, Los Angeles Institutional Review Board. Study 3: All procedures were approved by the Rutgers University Institutional Review Board and all participants provided written informed consent. Study 4: All procedures were approved by the University of California, San Francisco Committee on Human Research and all participants provided written informed consent. Study 5: All procedures were approved by the University of California, Los Angeles Institutional Review Board and all participants provided written informed consent.

### Study 1 – Identifying Items and Predictive Utility

#### Aims

Study 1 aimed to develop an initial set of items to assess the three complaints that characterize reward-based eating. A second aim was to examine the association between a novel self-report measure of RED and BMI in a large sample of men and women that includes a range of BMI. Finally, to explore the association between RED and key indicators of persistent obesity, we examined the relationship between RED and the timing of onset of overweight and weight fluctuation. We chose to develop this index of RED in the context of a prospective study following lean to obese individuals that used multiple eating measures – the Relationships of Energy and Nutrition to Obesity (RENO) Diet Heart Study (RDHS).

#### Procedures

The Institutional Review Board of the University of Nevada approved all study procedures and all participants provided written informed consent. The primary intent of the parent study (RDHS) was to examine predictors of and relations between weight fluctuations and CVD risk. Details of recruitment and study design for RDHS are described elsewhere [39]. Participants reported good health (no major illnesses or medications), no history of hospitalization for a psychological disorder in last five years, and no major depression. Participants were assessed at baseline and prospectively in their natural setting over eight years. We conducted analyses on the 327 participants who provided BMI data in the eighth year.

#### RED scale

In Study 1, we developed a set of items to capture the RED construct. Lack of control over eating: We selected three items that assessed feelings of control from the weight history questionnaire designed specifically for the RDHS [39]. These items assessed the extent to which respondents “*feel out of control eating on diet*,” “*feel out of control eating when not on a diet*,” and “*feel out of control eating in general.*” Internal consistency across these three items was excellent (Cronbach’s α = .93). Lack of satiation: We selected three items that assessed lack of satiation from the hunger subscale of the Three Factor Eating Questionnaire [40]. These included: “*I get so hungry that my stomach often seems like a bottomless pit*,” “*I am always hungry so it is hard for me to stop eating before I finish the food on my plate*,” and “*Sometimes things just taste so good that I keep on eating even when I am no longer hungry*.” Internal consistency across these three items was adequate (α = .56). Preoccupation with food: We included one item assessing preoccupation with food from the RDHS weight questionnaire that asked: “*Are you preoccupied with thinking about food?*” We standardized and averaged all responses. Internal consistency across all seven items was good (α = .80). As these items were on different response scales, we aggregated z-scores of each item to calculate a scale mean score.

#### Results

The baseline sample comprised 253 women and 255 men; 96% were White. The mean age was 44.71 years (*SD* = 14.1) and ranged from 19 to 77. We first tested to see whether the items were appropriate for factor analysis, and according to the Kaiser-Meyer-Olkin (KMO) Measure of Sampling Adequacy (.77) and Bartlett’s Test of Sphericity [*χ^2^* = 1628.97, *p*<0.001], they were. We conducted parallel analysis [41], [42] using the *fapara* [43] command in STATA (Version 13), we found two factors emerged. One appeared to capture both lack of control and the single-item preoccupation with food (explaining 47.15% of scale variance), and the other captured lack of satiation (explaining 16.8% of scale variance). At baseline the aggregated mean RED z-score was close to zero because it is a standardized score, 0.0042 (SD = 0.72) and ranged from –1.09 to 2.13. At baseline, the mean BMI was 26.94 (*SD* = 4.58) and ranged from 17.06 to 42.28. At year eight, the mean BMI was 27.32 (*SD* = 5.30) and ranged from 17.97 to 55.19. The sample was highly educated, ranging from 14 to 18 years of education, with a mean of 16.88 (*SD* = 0.88). RED and BMI were positively correlated at baseline (*r* = .38, *p*<.001). To test whether RED was longitudinally associated with changes in BMI from baseline to year eight, we regressed BMI assessed at year eight onto RED assessed at baseline, and also accounted for the baseline BMI assessment. As hypothesized, RED significantly predicted change in BMI (*β* = .05, *p* = .03).

Next, we tested whether RED associated with childhood onset of overweight and adult weight fluctuation. The onset of overweight was dummy coded such that 0 = never overweight, 1 = overweight by puberty, and 2 = overweight after puberty. Puberty was defined by self-report. ANOVA analysis revealed significant differences in RED z-scores depending on the overweight onset, *F*(2,426) = 30.69, *p*<.001. A post-hoc Tukey test revealed that all pairwise comparisons were statistically significant (*p’s*<.008). RED z-scores of individuals who were overweight by puberty (*M* = 0.29, *SD* = 0.67) had significantly greater RED z-scores (*p* = .008) than scores of individuals who became overweight after puberty (*M* = 0.03, *SD* = 0.67). RED z-scores of individuals who were overweight by puberty also had significantly greater RED z-scores (*p*<.001) than scores of individuals who were never overweight (*M* = –0.44, *SD* = 0.51). Individuals who were never overweight had significantly lower RED z-scores (*p*<.001) than individuals who became overweight after puberty as well.

We estimated weight fluctuation using the coefficient of variation of weight (the standard deviation of each participant’s eight weight values divided by their mean across the eight years). As predicted, RED was significantly associated with greater weight fluctuations (*β* = .04, *p*<.001). At one standard deviation above the mean on RED the average amount of weight fluctuation was 5.4%, compared to 3.8% among those at one standard deviation below the mean on RED.

### Study 2 – Scale Improvement and Content Validity

#### Aims

The aim of the prior study was to develop an initial set of items to tap into the reward-based eating construct, and indeed, the 7-item RED scale positively associated with BMI, BMI increase over 8 years, and greater weight fluctuation. The internal consistency of the items tapping lack of satiation was low, and we only had one item available to measure preoccupation with food. Thus, Study 2 first aimed to expand the scale. Our second aim was to establish content validity by having experts in eating behavior rate the resulting items with respect to how well they captured the three RED constructs.

#### Procedures

The University of California, Los Angeles Institutional Review Board approved all procedures. For our first aim of expanding the scale, we again inspected items published in well-established, high-quality existing scales that assess non-pathological eating patterns. The Three Factor Eating Questionnaire (TFEQ) [44], from which we drew items to capture lack of satiation in Study 1, contains items that tap two of the three complaints reported with regard to reward-based eating: disinhibition (i.e., lack of control) and hunger (i.e., lack of satiation). Although the Binge Eating Scale (BES) [24], contains many items with wording indicating eating pathology, select BES items assess normative preoccupation with food, the third RED-related complaint. Therefore, to improve the validity and reliability of the RED scale, we chose items from these two measures that best captured each respective symptom and added five questions that further tapped into each symptom (see Table 1). The final measure comprised 10 items (see Table 1).

**Table 1:** Items comprising the Reward-Based Eating Drive Scale.

Item	Complaint	Source	Factor Loading
1. I feel out of control in the presence of delicious food.	Lack of Control	Three-Factor Eating Questionnaire	.82
2. When I start eating, I just can’t seem to stop.	Lack of Control	Three-Factor Eating Questionnaire	.84
3. It is difficult for me to leave food on my plate.	Lack of Control	Three-Factor Eating Questionnaire	.64
4. When it comes to foods I love, I have no willpower.	Lack of Control	*Original*	.*77*
5. I get so hungry that my stomach often seems like a bottomless pit.	Lack of Satiation	Three-Factor Eating Questionnaire	.71
6. I don’t get full easily.	Lack of Satiation	*Original*	.64
7. It seems like most of my waking hours are preoccupied by thoughtsabout eating or not eating.	Preoccupation with Food	Binge Eating Scale	.81
8. I have days when I can’t seem to think about anything else but food.	Preoccupation with Food	Binge Eating Scale	.84
9. Food is always on my mind.	Preoccupation with Food	*Original*	.83

Note: One item, “Others may slow down when eating, but I tend to eat fast until I am done” was deleted due to low expert ratings.

For our second aim of examining content validity, we consulted 15 experts in the field of eating behavior, and asked them to complete an online survey in which they rated each RED scale item from 1 (*not at all*) to 5 (*very much*) in terms of how much each item captured its proposed construct (see Cappelleri et al. [20]).

#### Results

The average score across all items across all experts was 4.18. Nine of 10 items received a score at the midpoint (3.00) or higher. One item, “*others may slow down when eating, but I tend to eat fast until I am done*,” received a mean score of 2.53, indicating low content validity. We therefore dropped this measure for all future studies, resulting in a 9-item scale. With this item omitted, the average expert rating score across all items was 4.36.

### Study 3 – Testing Psychometric Properties

The primary aim of Study 3 was to test psychometric properties of the new, 9-item RED scale. Our second aim was to repeat exploratory factor analysis, as the new 9-item set of questions was substantially different from those tested in Study 1. A third aim was to test whether RED scale would be reliable across a broader range of sociodemographic groups and we therefore administered the RED scale to a large, online sample.

#### Sample & Procedures

The Institutional Review Board of Rutgers University approved all procedures and all participants provided written informed consent. The sample comprised 400 participants (50% female) drawn from Amazon’s Mechanical Turk (MTurk), an online micro-task market that harnesses the power of crowdsourcing to gather data. Prior studies investigating the reliability and validity of MTurk have found it to yield samples that are significantly more representative of the U.S. population than other internet survey tools, and that the data obtained is as reliable as those using traditional methods [45]. Furthermore, we followed standard recommendations [46] to increase data reliability, including duplicating questions and discarding participants who responded with non-matching answers. Each MTurk respondent was compensated $0.25 for completing the RED scale and questions about their weight.

#### RED scale

We used the 9-item scale resulting from Study 2. We queried participants’ agreement with each statement using a 5-point Likert scale ranging from 0 (*very false*) to 4 (*very true*).

#### Results

The mean RED score was 1.88 (*SD* = 0.71) and RED scores ranged from 0 to 4. Responses were normally distributed on a Q-Q plot and both skew (0.22) and kurtosis (0.41) were acceptable. Each individual item was normally distributed (all skew <0.55; all kurtosis <–1.15). Internal consistency for the measure was good (Cronbach’s α = .82). Because the new 9-item set of questions different substantially from that tested in Study 1, we again conducted parallel analysis to understand the scale’s factor structure. The KMO ( = .85) and Bartlett’s tests (*χ^2^* = 511.12, *p*<0.001) indicated the items were appropriate for factor analysis. We then submitted the items to parallel analysis [41], [42] using the *fapara* command [43] in STATA (Version 13). Although the item set was constructed to capture three reward-based eating constructs, this parallel factor analysis favored a single-factor solution, which explained 42% of the scale’s variance.

### Study 4 – Psychometric Properties and Comparison with Food Addiction

#### Aims

Study 4 comprised five aims. Our first aim was to confirm the single-factor structure of the RED scale that we observed in the previous study. Our second aim was to establish measurement invariance across Black and White race groups. Our third aim was to examine discriminant validity of the RED scale by examining its overlap and non-overlap with the Yale Food Addiction Scale (YFAS; [47]), which is designed to measure pathological levels of food addiction. Although we conceptualize the RED scale as a measure of reward-based overeating like the YFAS, the RED scale should assess a wider range of non-pathological compulsive eating, not addiction *per se*. We therefore expected a significant but moderate correlation between the RED scale and the YFAS. Our fourth aim was to test whether the RED scale or YFAS showed larger associations with BMI. Finally, our fifth aim was to again investigate further psychometric possibilities for the RED scale. To do so, we examined the psychometric properties of the RED administered as a 3-point Likert response scale (0–2).

#### Sample & Procedures

The Committee on Human Research of the University of California, San Francisco approved all procedures and all participants provided written informed consent. Because we were testing RED invariance across Black and White race, we chose a well-characterized cohort of women comprising Black and White race, in which recruitment was designed to minimize socioeconomic status differentials between the two races. Eighty randomly selected women from the Richmond, CA cohort of the National Heart Lung and Blood Institute Growth and Health Study (NGHS) participated in this study. NGHS was originally conducted in three US sites enrolling equal numbers of Black and White girls. NGHS began when the girls were 9–10 years old and continued annual assessments until they were 19–20 years old. Extensive information on this study and sample are available elsewhere [48]. The current study drew from a follow-up assessment we conducted in a randomly selected subset of NGHS participants at age 32. In the follow-up study, participants completed the RED scale and self-reported their weight measured on a scale mailed to them at home. Study staff conducted a home visit with 20 women to determine whether the self-reported weights and heights provided by the participants were accurate and reliable. The results for re-weighing the women at a home visit with a professional scale resulted in a perfect Spearman correlation for weight (*ρ* = 1.0), with a mean difference of 0.5 pounds, and an almost perfect correlation for BMI (*ρ* = 0.98), with a mean difference of 0.28 BMI units.

#### Results

The average BMI was 31.81 (*SD* = 9.84) and ranged from 16.97 to 52.12. The average RED score was 0.40 (*SD* = 0.36), and ranged from 0 to 1.89.

*Factor structure:* The KMO (.80) and Bartlett’s tests (*χ^2^* = 245.21, *p*<0.001) indicated factor analysis was appropriate. We used confirmatory factor analysis (CFA) via the *sem* command in STATA (version 13) using maximum likelihood estimation to confirm the single-factor structure [49]. The unidimensional model fit indices were unacceptable (Root Mean Squared Error of Approximation [RMSEA] = .12, 90% confidence interval (CI) = .08, .16]; Comparative Fit Index [CFI] = .88). Inspecting the modification indices revealed that allowing the error terms of two items (“*I have days when I can’t seem to think about anything else but food*” and “*Food is always on my mind*”) to covary would improve model fit. Given the similarity in these items, allowing these error terms to covary was theoretically plausible. Doing so yielded RMSEA = .09, CI = .04, .14 and CFI = .92. As Study 1 indicated a 2-factor solution, we also tested a 2-factor CFA. This yielded similarly unacceptable model fit indices (RMSEA = .12, CI = .08–.16, CFI = .87). Modification indices again indicated that allowing the two above items’ error terms would improve model fit. Doing so yielded almost identical fit indices as the unidimensional solution, RMSEA = .09, CI = .04–.14, CFI = .92. Given the almost identical fit between the 1-factor versus 2-factor solution, we believe that the single factor solution is the most parsimonious solution. Although the model fit indices were not ideal for either solution, the confidence interval of the RMSEA included.05 and the CFI was acceptable as it was greater than .90 [50].

*Race invariance:* Next, to test measurement invariance [51] across Black and White race, we compared two multigroup models using the *ginvar* command in STATA (Version 13). The first model was an unconstrained model in which no parameters were constrained for either the Black or White subgroups. The second model tested metric invariance by constraining the factor loadings of each group to be equal. If the resulting change in *χ^2^* fit with the given degrees of freedom from the second invariant model was non-significant according to the likelihood ratio test, this indicated metric invariance. Indeed, the *χ^2^* difference was non-significant, *χ^2^* difference (8) = 1.95, *p* = .98, indicating metric invariance, i.e., that the factor loadings were equal across Black and White individuals.

*Comparison to YFAS:* Gearhardt and colleagues [47] intend the YFAS to be used as a diagnostic measure, and 10 participants (12.5%) met the criteria for food addiction. However, we also alternatively considered a continuous measure computed as the total number of symptoms endorsed: The mean symptom count YFAS score was 2.20 (*SD* = 1.64) and ranged from 1 to 7. Internal consistency of the RED scale was again good (Cronbach’s α = .81), normally distributed according to a Q-Q plot, acceptable in skew (1.18), but platykurtic (2.35), likely due to the response scale (0–2) used in this study. Internal consistency of the YFAS was good (Cronbach’s α = .90). The RED scale and the YFAS, as expected, were significantly but moderately correlated (*r* = .50, *p* = .001), sharing 25% variance, therefore capturing overlapping but not identical constructs. Both the RED scale and the YFAS (the latter computed both as diagnosis and symptom count) were associated with BMI (RED: *r = *.34, *p* = .003; YFAS diagnosis: *r* = .65, *p*<.001; YFAS symptom count: *r* = .31, *p* = .006). However, after accounting for YFAS diagnosis, the RED scale was still significantly associated with BMI (*β* = 0.31, *p* = .02), whereas after accounting for the RED scale, YFAS diagnosis was no longer significantly associated with BMI (*β* = 0.09 *p* = .50). Similarly, after accounting for YFAS symptom count, the RED scale was still significantly associated with BMI (*β* = 0.26, *p* = .05), whereas after accounting for the RED scale, YFAS symptoms were no longer significantly associated with BMI (*β* = 0.17 *p* = .20). RED scores did not differ by race, *F*(79) = 0.61, *p* = .43, and race did not significantly interact with BMI to predict RED scores (*b* = –0.35, *p* = .38).

### Study 5 –Psychometric Properties and Comparison with Power of Food Scale

#### Aims

Study 5 comprised four aims. Our first aim was to confirm whether the factor structure of the RED was a single- or two-factor scale. Our second aim was to establish invariance across gender and age groups. Our third aim was to examine discriminant validity of the RED scale in relation to the PFS, which is a similar measure that captures hedonic drive for food [20], [21]. Our final aim was to determine whether RED or PFS showed larger associations with BMI.

#### Sample & Procedures

The University of California, Los Angeles Institutional Review Board approved all procedures. We used the MTurk platform to collect the data, and again followed standard recommendations [46] to increase data reliability. Each MTurk respondent was compensated $2.00 for completing the battery of questionnaires. Given the platykurtic nature of the 0–2 response scale observed in Study 4, we returned to the 5-point 0–4 Likert response scale.

The sample consisted of 381 individuals drawn from MTurk, and was 48% female, 51.7% male, and 0.3% “other.” The average BMI of the sample was 28.06 (*SD* = 7.22), and ranged from 14.47 to 67.54. Given this low minimum BMI, we confirmed the plausibility of the BMI range according to standards from the National Health Survey (Miller, 2003). The average age was 32.96 (*SD* = 11.52), and ranged from 18 to 81.

#### Results

The average RED score was 1.70 (*SD* = 0.95) and ranged from 0 to 4. Internal consistency of the RED scale was excellent (Cronbach’s α = .92) and skewness (0.21) and kurtosis (–0.69) were low. The items were appropriate for factor analysis according to the KMO (.91) and Bartlett’s Test of Sphericity [*χ^2^* = 2168.77, *p*<0.001]. CFA analyses revealed patterns very similar to Study 4. The unidimensional solution (RMSEA = .18, CI = .16–.19, CFI = .86) fit was not acceptable, and modification indices again indicated that model fit would be improved by allowing the error terms of the same two items (“*I have days when I can’t seem to think about anything else but food*” and “*Food is always on my mind*”) plus another similar item, “*It seems like most of my waking hours are preoccupied by thoughts about eating or not eating*” to covary. This resulted in slightly worse RMSEA (.09) than in Study 4, but excellent CFI compared to Study 4 (.97). Factor loadings of each item appear in Table 1.

To establish invariance across gender and age, we conducted analogous analyses to Study 4, first testing unconstrained models and then constraining factor loadings of the groups to be equal. In the case of age, the median was exactly 30 years. We created a median split and tested invariance across those younger than 30 and those older than 30. For gender, the scale demonstrated metric invariance, *χ^2^* difference (8) = 6.26, *p* = .62. For age, the scale also demonstrated metric invariance, *χ^2^* difference (8) = 1.69, *p* = .99.

The average PFS score was 2.85 (SD = 0.95) and ranged from 1 to 5. Internal consistency of the PFS was also excellent (Cronbach’s α = .94). To examine the RED in comparison with the PFS, we first examined zero-order correlations between the two measures. As might be expected, they were significantly correlated (*r* = .70, *p*<.001). Both were significantly related to BMI (RED: *r* = .35, *p*<.001; PFS: *r* = .26, *p*<.001). We then entered the RED and the PFS simultaneously in multiple regression analyses. RED remained a significant predictor of BMI (*β* = 0.16, *p* = .04), whereas the PFS did not (*β* = 0.04, *p* = .63).

## Discussion

Across five studies, we assessed eating characterized by three reward-related eating complaints – lack of control over eating, lack of satiation, and preoccupation with food. We developed a 9-item index that ultimately captured reward-based eating drive as a single factor and was reliable (internally consistent). It also demonstrated invariance across gender, age, and Black/White race. We tested a 3-point Likert scale (0–2), which yielded platykurtic results, and tested a 5-point (0–4 response) Likert scale, which yielded better psychometric properties that more closely approximated a normal distribution.

We found preliminary evidence that endorsing a high reward-based eating drive as indexed by the earliest version of the RED scale was positively associated with BMI and predictive of change in BMI over eight years. Additionally, this version of the RED scale associated with indicators of more life-long obesity as indicated by pre-puberty overweight onset, although time of puberty onset was based on retrospective recall and therefore subject to recall bias. It was also associated with more frequent weight fluctuation measured over the 8 years, possibly indicating repeated dieting and failures at maintaining weight loss. The RED scale may therefore be useful in a screening context to identify (1) people at risk of overweight, and (2) overweight people who are at risk for further weight gain or yo-yo dieting.

The RED scale and Power of Food Scale were highly related but the RED scale had an independent relation to BMI, unlike the PFS, when both were tested in one model. We found analogous results with the Yale Food Addiction Scale. Although the RED scale is similar to these other recently-developed self-report measures, the data presented here suggest the RED scale correlates both concurrently and prospectively with BMI, indictaing that it may be useful in identifying a stable behavioral phenotype. We suggest that in addition to its unique ability to correlate with and predict BMI, the RED scale adds a necessary component to these existing scales in several ways. The PFS is a highly internally-valid scale that captures the psychological impact of food in the environment, and carefully delineates between food available, food present, and food tasted. Unlike the RED scale, however, its focus is on the effect of the environment on the individual, whereas the RED scale focuses on internal cognitions and behaviors. Unlike the Yale Food Addiction Scale, which is designed to diagnose clinical levels of food addiction, the RED scale appears to capture information that spans a non-pathological continuum of reward-based eating. Data presented here demonstrate that the RED scale can be appropriately used to assess the more normative reward-based eating in non-clinical samples.

From the current studies, we cannot conclusively identify which comes first – the reward-based eating or higher BMI. Obesity, and in particular insulin resistance, might drive symptoms of reward-based eating [52], as insulin dampens reward activity [53] and low insulin enhances both reward [54] and opioid activity [55], [56]. We did, however, find that scoring higher on reward-based eating was associated with increases in BMI over eight years, prospectively, which suggests that the reward-based eating may have come first. Furthermore, these relationships are likely not mutually exclusive in that both reward drive and the physiological state of obesity, mainly insulin resistance, can both stimulate further overeating [57]. However, both reward-based eating and obesity could be influenced by third variables such as childhood stress.

In future studies, longitudinal designs could assess reward-based eating vulnerability in young children (prior to any onset of metabolic dysregulation) and parents, following both prospectively to examine both parental transmission and temporal sequence of developmental trajectories of behaviors and obesity. Although we found consistent relationships across a number of samples with varying demographic characteristics, each study was not without limitation in its generalizability. In Study 1, for example, the sample was highly educated and predominantly White, and the question set was rudimentary. Further work is needed to establish discriminant validity of the RED scale with other commonly-used scales assessing similar constructs. Future research should examine multiple measures tapping aspects of eating in the context of a single study to clarify if and how measures are concurrently and prospectively associated both with each other and with biomarkers of metabolic health, as well as how these measures change in response to weight-loss interventions. We did not screen for eating disorder diagnoses or symptomatology, and therefore cannot speak to whether or how the RED scale operates differentially in this pathological population.

We note that the RED scale is shorter than any of the other eating measures cited in this paper. The brevity of the RED scale lends itself well to research and with further validation, for use in clinical settings as a potential screening instrument to identify those at risk of gaining weight. Given that the literature on food and reward processes has developed rapidly within the last decade or so, the RED scale incorporates a more focused perspective reflecting current neuroscience that meshes with clinicians’ and individuals’ experiences, and represents a potentially effective new tool in the treatment toolkit.

Lastly, we contrast a screener like the RED scale to the types of careful experimental assessments that can tap the distinct processes of reward sensitivity and pathology. The RED scale was derived through clinical observations – thus it reflects what is accessible to conscious processing and explicit memory. It identifies a clinical phenotype rather than the many core neurobehavioral mechanisms of reward, some of which may or may not be tightly linked to self-report measures. While the RED scale is based on what people commonly notice and report, there are underlying neurocognitive mechanisms of reward that can be measured more directly. Future research that assesses laboratory-based measures of reward processes should clarify associations between laboratory-based measures and self-report measures of reward-based processes in a more detailed and thorough assessment, to the extent that people can report on them. The NIMH Research Domain Criteria (RDoC) offers some guidance relevant to overeating. The positive valence domain includes many processes such as approach motivations (e.g., craving, goal-directed behaviors), initial responsiveness to reward attainment (e.g., how good food tastes initially), sustained responsiveness (e.g., level of satiety), reward learning (e.g., cue triggered eating), and habit (e.g., automatic consummatory responses). The RED scale measures some of these, albeit indirectly. For example, RED scale satiety items measure lack of sustained responsiveness. Preoccupation with food (e.g., “food is always on my mind”) has been highly associated with food cravings (e.g., [58]). Lack of control over eating likely taps into several core motivational and executive functioning processes: For example, the item “*I feel out of control in the presence of delicious food*” may reflect both high reward drive (willingness to work for reward) and poor ‘brakes’ on unwanted behavior. The ability to inhibit an unwanted behavior is part of the cognitive control domain of RDoC, and is measured directly as response inhibition or high impulsivity. The resulting overeating and feeling out of control is thus likely reflecting a combination of both high reward reinforcement and poor cognitive control, the combination of the two has been labeled “reinforcement pathology” [59]. Thus, while the screener may have predictive value, and may be related to reward-based drive crudely, it is not a substitute for more intensive longer behavioral and self report measures that can deconstruct aspects of neurocognitive processes.

## Conclusion

We speculate that the reward-based eating drive scale explored here may prove a useful tool to identify a target population for customized obesity treatment or prevention. Higher reward-based eating drive was associated with higher BMI and more persistent obesity. Individuals with higher scores on the RED scale may benefit from adjuvant treatment components that take into account the compulsive nature of eating. The underpinnings of reward-based eating drive represent a new and underdeveloped area in obesity assessment and treatment, and researchers have called for incorporating weight-loss strategies that take the rewarding nature of eating into account [60]. We hope the short RED screening tool presented here might help researchers and treatment providers to better identify and understand reward-based eating before it leads to further decline in metabolic health, or in some cases, eating pathology. Given the heterogeneity of the causes of and the difficulties in treating obesity, targeting basic reward-based eating drive rather than BMI alone may lead to more effective prevention and treatments.
